# Migration of T Cells on Surfaces Containing Complex Nanotopography

**DOI:** 10.1371/journal.pone.0073960

**Published:** 2013-09-12

**Authors:** Keon Woo Kwon, Hyoungjun Park, Junsang Doh

**Affiliations:** 1 Department of Mechanical Engineering, Pohang University of Science and Technology (POSTECH), Pohang, Republic of Korea; 2 School of Interdisciplinary Bioscience and Bioengineering (I-Bio), Pohang University of Science and Technology (POSTECH), Pohang, Republic of Korea; University of California, Riverside, United States of America

## Abstract

T cells navigate complex microenvironments to initiate and modulate antigen-specific immune responses. While recent intravital microscopy study revealed that migration of T cells were guided by various tissue microstructures containing unique nanoscale topographical structures, the effects of complex nanotopographical structures on the migration of T cells have not been systematically studied. In this study, we fabricated surfaces containing nanoscale zigzag structures with various side lengths and turning angles using UV-assisted capillary force lithography and motility of T cells on zigzag patterned surfaces was studied. Motility of T cells was mostly affected by the turning angle, not by the side length, of the zigzag structures. In particular, motility behaviors of T cells near interfaces formed by turning points of zigzag patterns were significantly affected by turning angles. For obtuse turning angles, most of the T cells smoothly crossed the interfaces, but as the turning angle decreased, a substantial fraction of the T cells migrated along the interfaces. When the formation of lamellipodia, thin sheet-like structures typically generated at the leading edges of migrating cells by actin polymerization-driven membrane protrusion, was inhibited by an Arp2/3 inhibitor CK-636, a substantial fraction of T cells on those surfaces containing zigzag patterns with an acute turning angle were trapped at the interfaces formed by the turning points of the zigzag patterns. This result suggests that thin, wide lamellipodia at the leading edges of T cells play critical roles in motility of T cells in complex topographical microenvironments.

## Introduction

T cells are immune cells playing a central role in antigen-specific immune responses. To successfully mount antigen-specific immune responses, T cells must migrate to the right place and encounter their partners [1]. For example, they become activated by interacting with antigen-presenting cells presenting antigens specific for their T cell receptors in secondary lymphoid organs such as a spleen and lymph nodes, and they perform effector functions by contacting pathogen-harboring cells or transformed cells in peripheral tissues. Therefore, how quickly T cells find their interaction partners may determine the overall efficacy of immune responses [1], [2].

Multi-photon microscopy performed over the last decade has allowed us to understand how T cells migrate in search for their interaction partners in vivo [3], [4]. Overall, they migrate rapidly with a peak velocity of 25 µm/min in a rather random fashion to maximize the scanning area [5]. At the same time, their motility is guided not only by soluble factors such as chemokines [6], but also by many cellular/extracellular structures such as collagen fibers [7], specialized lymph node stromal cells called fibroblastic reticular cells [8], and fibrous structures formed by infection [9], which typically have unique nanoscale topographical structures. While the effect of soluble factor on directional migration of T cells has been extensively studied using various in vitro model systems such as agarose gel [10], Boyden chambers [11], and microfluidic channels [12], relatively less attention has been paid to the effects of nanotopography on motility of T cells.

Recently, we investigated how motility of T cells is affected by nanoscale topographical structures mimicking fibrous structures of ECMs using polymer surfaces containing straight lines of nanoscale topographical structures [13]. Compared with epithelial and mesenchymal cells, which have been extensively studied using nanostructured surfaces [14]–[17], T cells exhibit a completely different mode of migration, so called amoeboid migration: T cells only weakly adhere to the substrates, generate weak traction forces and migrate 10–100 times faster than epithelial cells and fibroblasts [18]. As a result, the behavior of T cells on nanogrooved surfaces was different from that of epithelial/mesenchymal cells. While epithelial/mesenchymal cells aligned almost perfectly and migrated along the nanogroove direction, migration of T cells were close to a biased random walk with increasing directional persistence with increasing adhesiveness [13]. Lamellipodia, a thin sheet-like membrane protrusion, at the leading edge appeared to be guided toward the direction of the nanogrooves when adhesive substrates were used, but the role of lamellipodia on topography sensing of T cells has not been fully elucidated. Moreover, straight nanoscale ridge/groove structures may not fully represent the complex topographical structures T cells encounter in vivo.

To address the aforementioned issues, we fabricated surfaces containing nanoscale zigzag structures with various side lengths and turning angles, and then investigated the effects of these zigzag nanotopographical structures on the motility of T cells. The roles of lamellipodia in T cell migration on complex nanotopographical surfaces were studied by treating T cells with a pharmacological inhibitor targeting Arp2/3, a key regulator for lamellipodia formation [19], and comparing the motility of Arp2/3 inhibitor-treated T cells with that of untreated T cells.

## Results and Discussion

### Preparation of the Nanoscale Zigzag Structures

**Figure 1:**
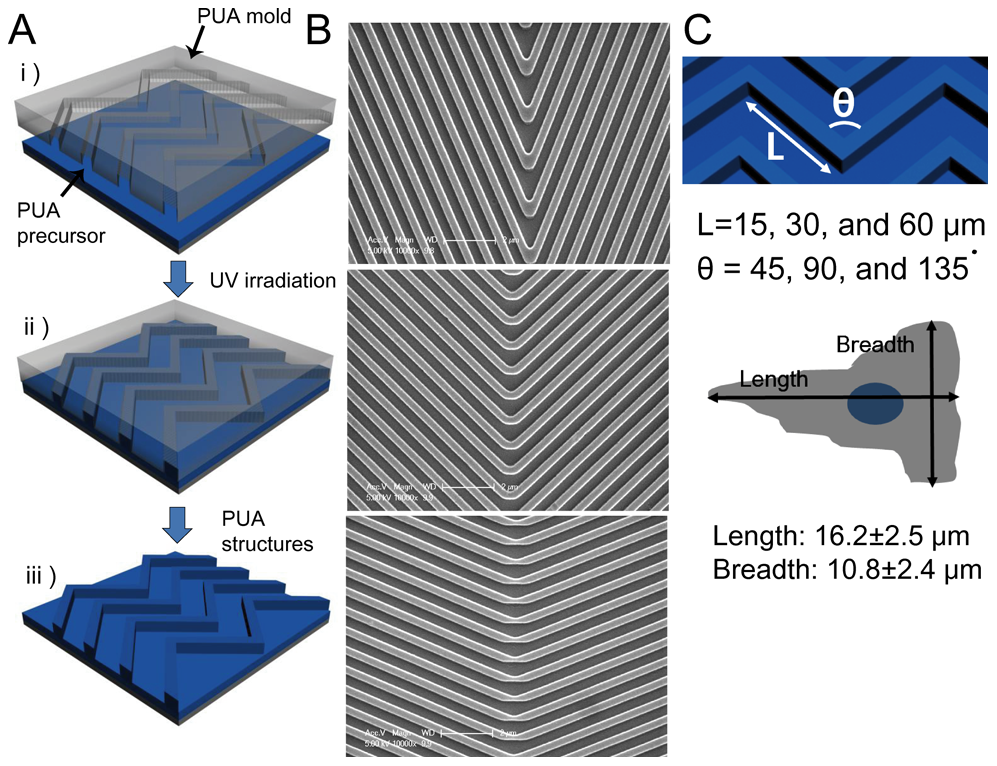
Fabrication of the nanoscale zigzag structures. (A) A schematic illustration of the fabrication of surfaces containing nanoscale zigzag structures. (B) SEM images of various zigzag structures. Top: side length (L) = 60 µm, turning angle (θ) = 45°; Middle: L = 60 µm, θ = 90°; Bottom: L = 60 µm, θ = 135°. (C) A schematic diagram of the nanoscale zigzag structures and dimensions (Top) and the average dimensions of T cells (Bottom) used in our experiments.

To study how the motility of T cells is affected by complex nanotopography, we fabricated nanoscale zigzag structures using UV-assisted capillary force lithography (CFL) with UV curable polymer PUA on glass coverslips as shown in Fig. 1A [20]. Scanning electron microscope images of successfully fabricated nanostructured surfaces are shown in Fig. 1B. The width of ridges, grooves, and the height of the nanoscale zigzag structures were 350 nm, 700 nm, and 300 nm, respectively, which mimic the dimensions of fibrous extracellular matrix (ECM) structures [17], [21]. Three different lengths of sides (L = 15, 30, and 60 µm) and three different turning angles (θ = 45°, 90°, and 135°) of nanoscale zigzag structures were fabricated (Fig. 1C). The average length and breadth of the T cells used for the experiments were 16.2 µm and 10.8 µm, respectively, close to the shortest length of the fabricated zigzag structures. Nanostructured PUA surfaces were coated with 10 µg/mL of ICAM-1, a ligand for lymphocyte function-associated antigen 1 (LFA-1) that is one of major integrins of T cells.

### Migration of T cells on the Surfaces Containing Zigzag Nanotopography Structures

**Figure 2:**
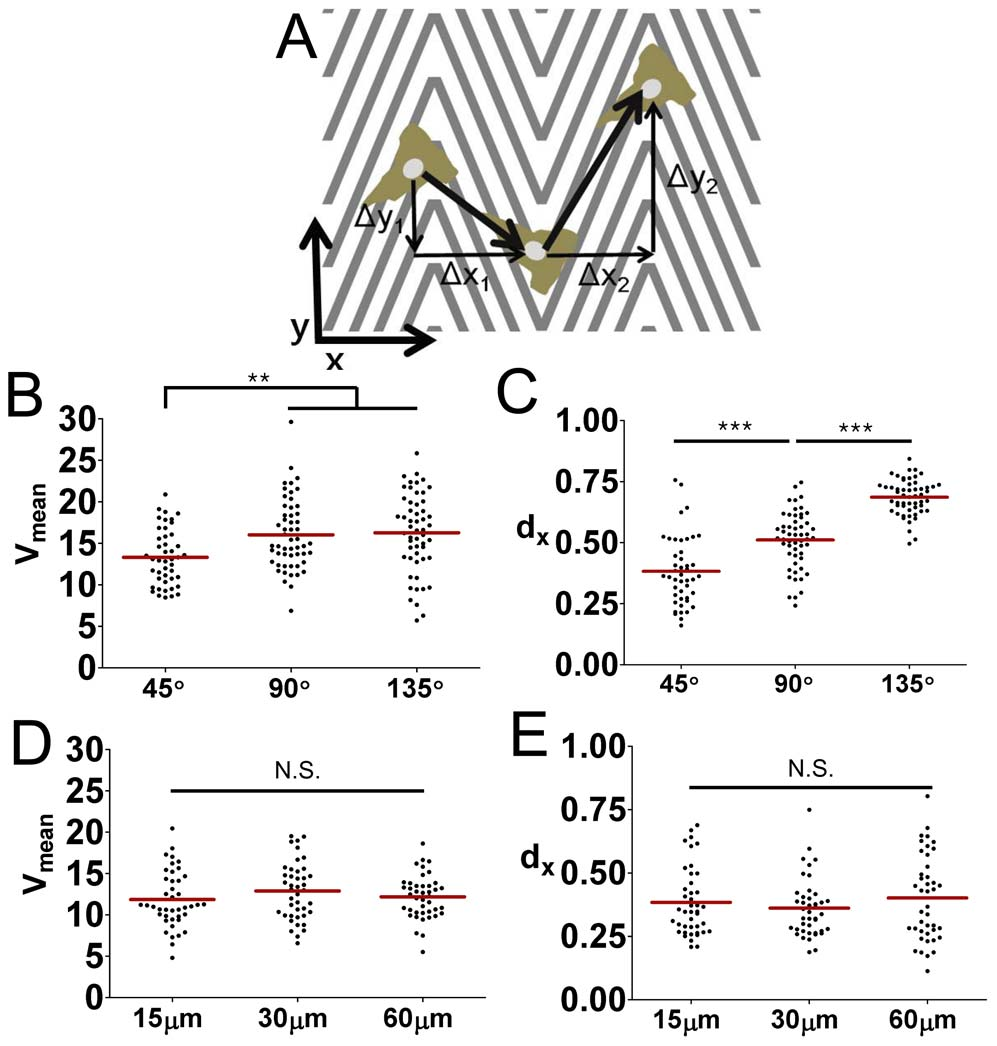
Effect of side lengths (Ls) and turning angles (θs) of the nanoscale zigzag structures on T cell migration. (A) A scheme of quantitative evaluation of the mean velocity (V_mean_) and directionality (d_x_) of T cell migration. (B, C) Effect of θ on the V_mean_ (B) and the d_x_ (C) values of T cells on the nanoscale zigzag structures with fixed L (60 µm, n = 45 for 45°; n = 54 for 90° and 145°). (D, E) Effect of L on the V_mean_ (D) and the d_x_ (E) of T cells on the nanoscale zigzag structures with fixed θ (45°, n = 42 for all cases). Data are representative of two independent experiments. (line: average, Mann-Whitney test, N.S.: not significant, **p<0.001, ***p<0.0001).

To quantitatively analyze the motility of the T cells on the nanoscale zigzag structures, zigzag patterns were mounted on a microscope stage with the orientation schematically shown in the left panel of Fig. 2A so that all the sides of the zigzag patterns will have identical angles, ± half of the turning angle θ, to y-axis. Then, cell movements were recorded by time-lapse microscopy, and the trajectories of individual T cells were tracked by image processing, and analyzed. Δx and Δy are displacements of T cells along the x- and y-axis, respectively. From the displacement information, mean velocity (V_mean_) and directionality of migration along the x-axis (d_x_) were calculated using the equations shown below:
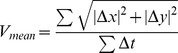


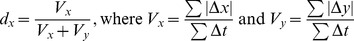



The d_x_ value is larger or smaller than 0.5 when T cells migration is biased either to the direction of x-axis or y-axis, respectively, and it is close to 0.5 if the T cell migration is not biased to any directions.

First, we assessed the effect of turning angle θ on the velocity and directionality of T cell migration. The V_mean_ and d_x_ values of T cells on zigzag patterns with fixed side length L = 60 µm and various turning angles were plotted in Fig. 2B and C, respectively. The average V_mean_ value of T cells on zigzag patterns with θ = 45° was significantly lower than that of T cells on zigzag patterns with θ = 90° or 135°, meaning that motility of T cells was hampered by nanoscale zigzag topographical structures with a sharp turning angle. The average values of d_x_ of T cells on the zigzag patterns with 45°, 90°, and 135° was 0.38, 0.51, and 0.69 respectively, meaning that the migration of T cells on the zigzag patterns with 45° and 135° of turning angles was biased along the y- and x-axis, respectively, while the migration the T cells on the zigzag patterns with 90° turning angle was not biased to x or y directions. These results indicate that T cell migration was guided by nanoscale topographical structures as previously demonstrated [13] because the sides of the zigzag patterns with 45° and 135° of turning angles are oriented toward y-axis and x-axis, respectively while sides of zigzag patterns with 90° of turning angles are not biased toward any axis. Then, we assessed whether the lengths of sides of the zigzag patterns affected the migration of T cells by using zigzag patterns with various Ls. Both V_mean_ and d_x_ values were not affected by L even at the sharpest turning angle (Fig. 2D and E). Indeed, similar results were obtained with all the turning angles examined (data not shown), meaning that the length of the sides of the zigzag patterns had minimal effect on T cell motility. Therefore, we fixed the value of L at 60 µm for the rest of the study and primarily focused on the effect of the turning angles.

**Figure 3:**
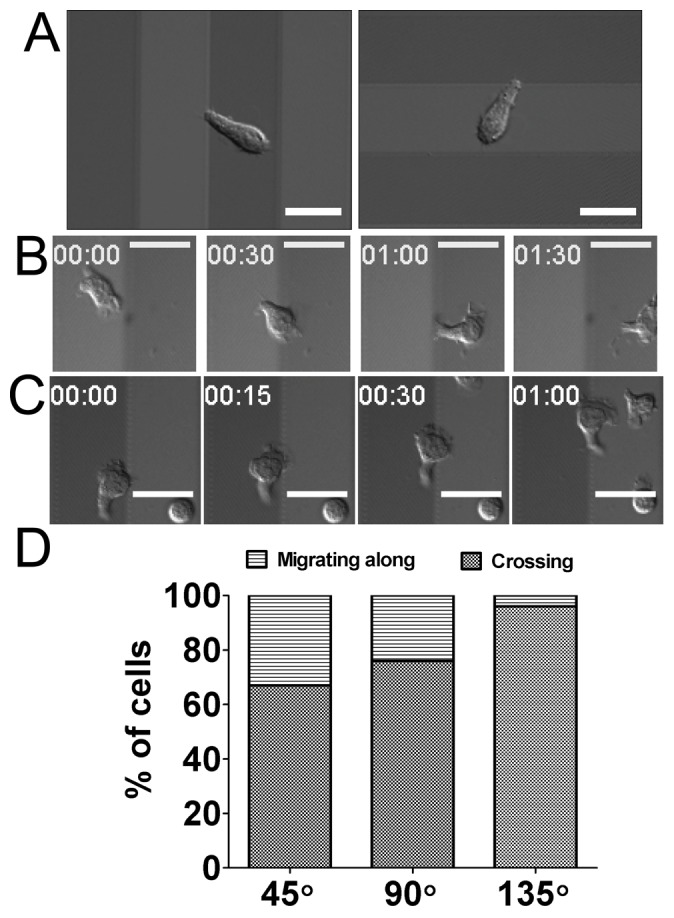
Motility behaviors of T cells at the interfaces formed by turning points of nanoscale zigzag structures. (A) A representative differential interference contrast (DIC) image of nanoscale zigzag structured surface with L = 30 µm and θ = 135° (left) and a DIC image of the same surface after 90° clockwise rotation (right). Scale bar = 20 µm. (B) Representative time-lapse DIC images of a T cell ‘crossing’ the interfaces of the nanoscale zigzag structures (L = 60 µm, θ = 45°). Time stamp = min:sec, Scale bar = 20 µm. (C) Representative time-lapse DIC images of a T cell ‘migrating along’ the interfaces of the nanoscale zigzag structures (L = 60 µm, θ = 45°). Time stamp = min:sec, Scale bar = 20 µm. (D) Effect of θ on motility behaviors of T cells at the interfaces formed by turning points (n = 44 for 45°; n = 55 for 90°; n = 54 for 145°). Data are representative of two independent experiments.

While overall motility and directionality of T cells can be described by V_mean_ and d_x_, they contain limited information about the local behaviors of T cells, in particular near the turning points of the zigzag patterns. Interfaces composed of turning points of zigzag patterns can easily be detected by differential interference contrast (DIC) mode of imaging, which uses polarized light, because the amount of transmitted light differs depending on the angle between the polarized light and the sides of the zigzag patterns. As a result, clear boundaries were visible at interfaces formed by the turning points of the zigzag patterns, and when the surface containing the zigzag surface was rotated 90° clockwise, the dark and bright areas were altered (Fig. 3A). When we carefully examined the T cells entering interfaces defined by the turning points of zigzag patterns, we observed two distinct populations of T cells. Some T cells crossed the interface with minimal residence time at the interfaces denoted as ‘crossing’ (Fig. 3B and Movie S1), and some T cells migrated along the interfaces denoted as ‘migrating along’ (Fig. 3C and Movie S2). The percentage of each population was measured for zigzag patterns with different turning angles and plotted in Fig. 3D. Most of the T cells on the zigzag patterns with 135° turning angle crossed the peaks smoothly, but as the turning angles became sharper, more and more T cells migrated along the interfaces, and about 30% of the T cells on the zigzag patterns with 45° turning angle migrated along the peaks parallel to the y-axis.

### Roles of Lamellipodia on Motility Behavior of T cells on the Surfaces Containing Zigzag Nanotopography Structures

**Figure 4:**
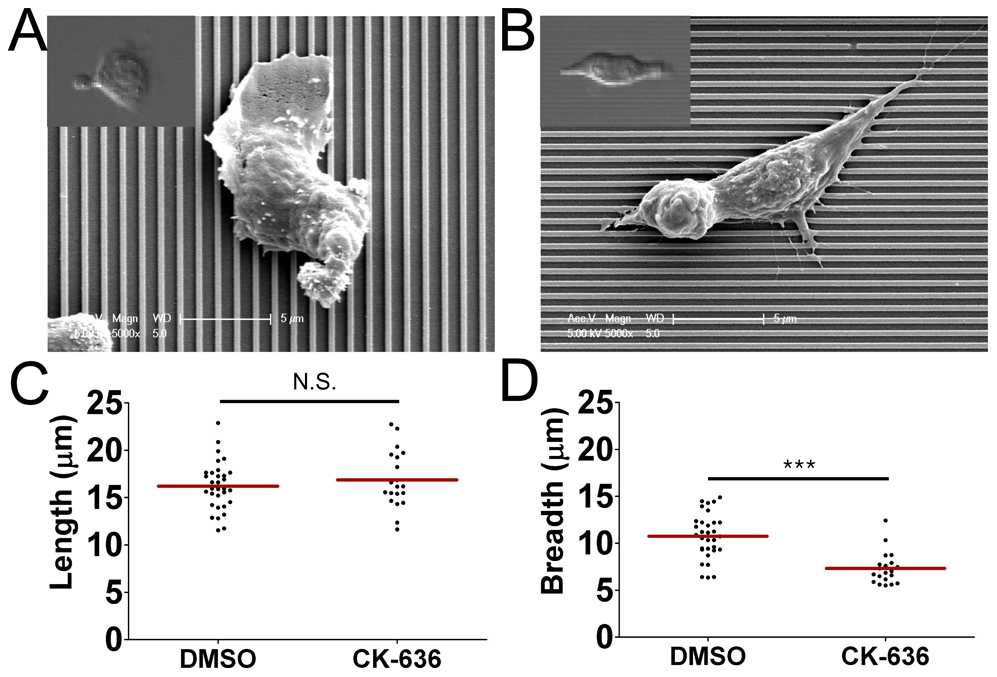
Effect of Arp2/3 inhibitor treatment on morphology of T cells. (A, B) Representative SEM and bright field (inset) images of T cells on the 350 nm ridges/700 nm grooves either treated with DMSO (A) or CK-636, an Arp2/3 inhibitor (B). (C, D) Effect of CK-636 treatment on length (C) and breadth (D) of T cells on the 350 nm ridges/700 nm grooves (n = 32 for DMSO; n = 20 for CK-636). (line: mean, Mann-Whitney test, N.S.: not significant, ***p<0.0001).

Migrating T cells exhibit a characteristic hand-mirror shape with wide lamellipodia at the leading edge (Fig. 4A). In our previous study, we demonstrated that actin polymerization driven leading edge protrusion is critical for contact guidance of T cells [13], but the role of the lamellipodia, which form at the leading edges of migrating T cells, on nantopography-guided migration of T cells has not yet been elucidated. To address this, we inhibited Arp2/3, which nucleates actin branching to form lamellipodia [19], using CK-636, a recently developed pharmacological inhibitor [22]. To assess whether CK-636 inhibits formation of lamellipodia in T cells, we fixed CK-636-treated T cells on nanostructured surfaces and examined their ultrastructures using SEM. For a control, T cells treated with DMSO, a carrier used to dissolve CK-636, were also examined. While the DMSO-treated T cells exhibited characteristic hand mirror-shaped morphology with wide and thin sheet-like lamellipodia at the leading edges (Fig. 4A), the CK-636-treated T cells exhibited elongated morphology with sharp pseudopodia at the leading edges (Fig. 4B). When we quantitatively analyzed length and breadth of the T cells, the length of the CK-636-treated T cells were comparable to those of the DMSO-treated T cells (Fig. 4C), while the breadth of the CK-636-treated T cells was about 30% less than that of DMSO-treated T cells (Fig. 4D).

**Figure 5:**
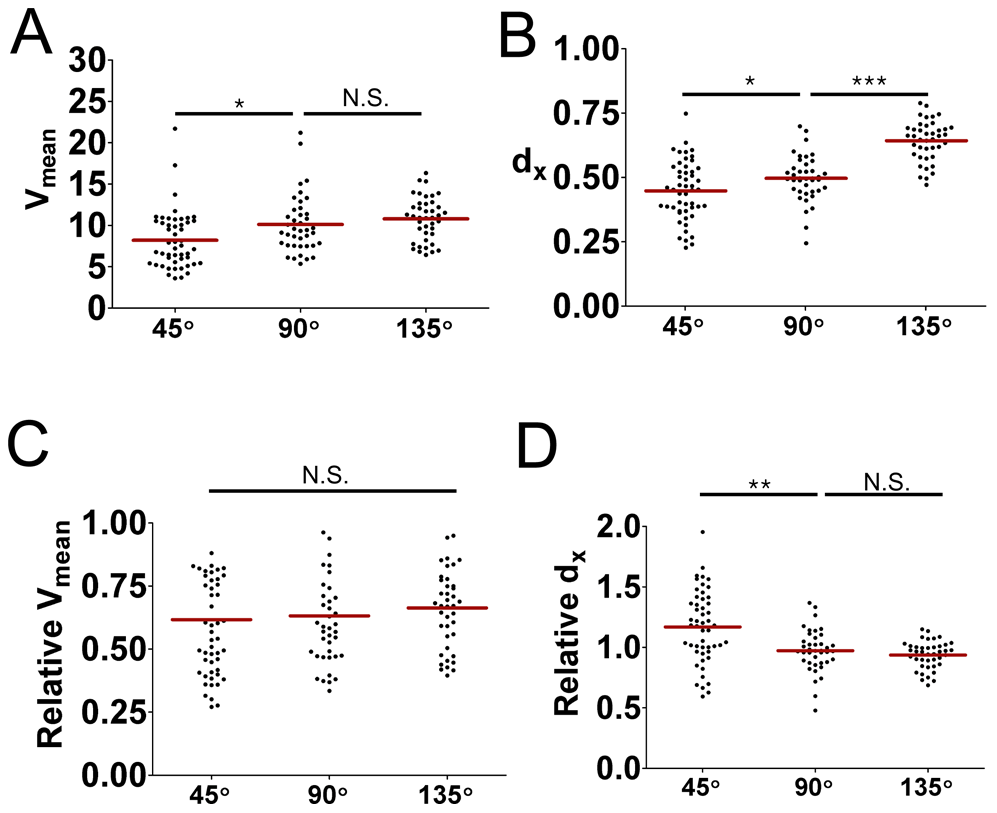
Effect of Arp2/3 inhibition on velocity and directionality of T cell migration. (A, B) Effect of Arp2/3 inhibition on the V_mean_ (A) and the d_x_ (B) values of T cells on the nanoscale zigzag structures with θ = 45°, 90°, and 135° (n = 51 for 45°; n = 40 for 90°; n = 41 for 145°). (C) Relative V_mean_ values of T cells defined by the ratio between V_mean_ values of CK-636-treated T cells and the average of V_mean_ values of DMSO-treated T cells. (D) Relative d_x_ values of T cells defined by the ratio between d_x_ values of CK-636-treated T cells and the average of d_x_ values of DMSO-treated T cells. Data are representative of two independent experiments. (line: mean, Mann-Whitney test, N.S.: not significant, *p<0.05, **p<0.001, ***p<0.0001).

With this clear inhibition of lamellipodia formation at the leading edge by CK-636-treated T cells, we next performed migration assays of CK-636-treated T cells on the surfaces containing nanoscale zigzag structures with various turning angles. The V_mean_ and d_x_ values of CK-636-treated T cells were calculated and plotted in Fig. 5A and 5B, respectively. To compare the motility of CK-636-treated T cells with the untreated T cells, the relative V_mean_, or d_x_, values were defined by the ratio between V_mean_, or d_x_, values of CK-636-treated T cells and the average of V_mean_, or d_x_, values of untreated T cells on the same type of surfaces. Calculated relative V_mean_ and d_x_ values of CK-636-treated T cells were plotted in Fig. 5C and 5D, respectively. Similar to the case of untreated-T cells (Fig. 2B), the average V_mean_ value of the CK-636-treated T cells on surfaces with θ = 45° was significantly lower that of CKC-636-treated T cells on surfaces with θ = 90° and 135° (Fig. 5A). In addition, the relative V_mean_ value of CK-636-treated T cells was slightly above 0.6 for all the turning angles examined (Fig. 5C), meaning that CK-636 treatment significantly reduced the velocity of T cells on the surfaces regardless of turning angles. In contrast, normalized d_x_ values of CK-636-treated T cells on zigzag surfaces with θ = 45° was about 1.2 while normalized d_x_ values of CK-636-treated T cells on zigzag surfaces with θ = 90° and θ = 135° were close to 1, indicating that abrogation of lamellipodia formation at the leading edges of T cells by the treatment of CK-636 had profound impact on the directionality of T cells on zigzag patterns with an acute turning angle, but minimal effect for T cells on zigzag patterns with right or obtuse turning angles.

**Figure 6:**
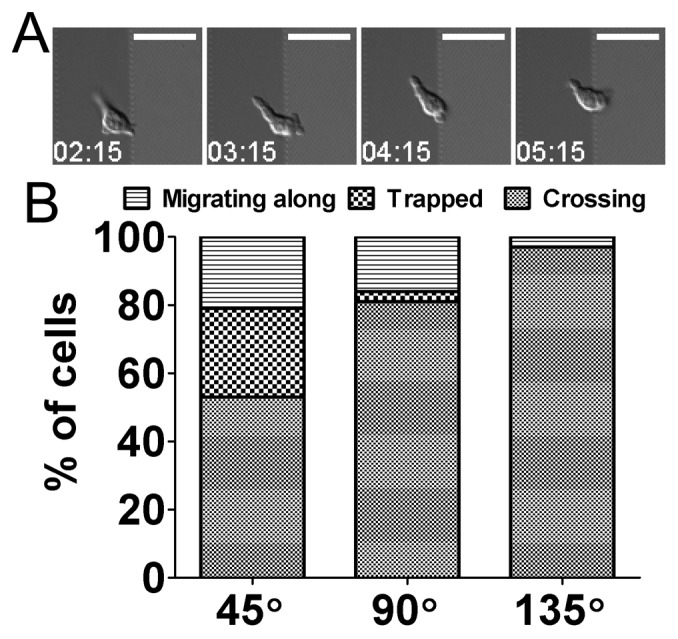
Effect of Arp2/3 inhibition on motility behavior of T cells at the interfaces formed by turning points of nanoscale zigzag structures. (A) Representative time-lapse DIC images of a CKC-636-treated T cell ‘trapped’ at the interfaces of formed with turning points of the nanoscale zigzag structures (L = 60 µm, θ = 45°). Time stamp = min:sec, Scale bar = 20 µm. (B) Effect of θ on motility behaviors of CKC-636-treated T cells at the interfaces formed by turning points (n = 55 for 45°; n = 38 for 90°; n = 31 for 145°). Data are representative of two independent experiments.

To gain further information, we analyzed the behaviors of CK-636-treated T cells near interfaces where the direction of the sides changed. Interestingly, a few T cells stayed at the interfaces for more than two minutes with minimal net translocation longer than 2 min. (Fig. 6A and Movie S3). This population of T cells was denoted as ‘trapped’, and T cells encountering interfaces formed with turning points of zigzag patterns were classified into three categories and plotted in Fig. 6B. As expected, the behavior of CK-636-treated T cells on zigzag patterns with θ = 90° or 135° were not much different from those of untreated T cells: more than 95% of T cells crossed the interfaces when θ = 135° while the population of T cells migrating along the interfaces increased when θ became smaller. In contrast, more than 25% of CK-636-treated T cells were trapped at the interfaces when θ = 45°.

These results suggest that wide and thin lamellipodia formed at the leading edge of T cells are important for maintaining the motility of T cells on complex topography. Coordinated membrane protrusion and focal adhesion formation via lamellipodia have recently been shown to be important for directed migration of fibroblasts toward biochemical cues [23], [24]. Our previous study of T cell migration on straight nanogrooves coated with ICAM-1 also showed that T cells generated aligned protrusion of lamellipodia toward the direction of nanogrooves [13]. Thus coordinated leading edge protrusion via lamellipodia may also be important for nanotopography-guided migration of T cells. In addition to coordinating leading edge protrusion of migrating cells to augment directed migration of cells, lamellipodia may also promote motility of cells under complex nanotopogrphical microenvironments, which may be formed by interwoven fibrous bundles of ECMs in vivo, by allowing smooth direction change as demonstrated in this study.

## Conclusions

In summary, surfaces containing various nanoscale zigzag structures were fabricated by CFL and the effects of complex nanotopography on the motility of T cells were studied. Motility of T cells was mostly affected by the turning angle, not by the side length, of the zigzag structures. T cells on zigzag patterns with an acute turning angle exhibited significantly reduced migration speed and altered migration direction compared with T cells on zigzag patterns with right or obtuse angles. Lamellipodia formation at the leading edges of migrating T cells could be inhibited by treating T cells with CK-636, a pharmacological inhibitor targeting Arp2/3. Overall, CK-636-treated T cells exhibited reduced velocity compared with untreated T cells. In particular, a substantial fraction of CK-636-treated T cells on zigzag patterns with an acute turning angle were trapped near the interfaces formed by the turning points of zigzag patterns, suggesting that lamellipodia play essential roles in rapid migration of T cells under complex topographical microenvironments.

## Materials and Methods

### Fabrication of Nanoscale Zigzag Structured Surfaces

A silicon master of nanoscale zigzag structures was fabricated at the Korea Advanced Nano Fab Center (KANC). To fabricate nanoscale structures on large areas of silicon wafer, Krypton-Fluoride (KrF) stepper was used in photolithography. Nanostructures on the silicon master were replicated using poly(urethane acrylate) (PUA) by UV-assisted capillary force lithography (CFL), and by replicating the PUA nanostructured surfaces one more time on thin glass coverslips by CFL. In this way, nanostructures identical to the original silicon master were formed on coverslips [20]. The glass coverslips were cleaned using ethanol and deionized (DI) water and dried in a vacuum oven. Next, the dried coverslips were coated with adhesion promoter (Minuta Tech) and baked in the oven at 120°C for 15 min. The PUA precursor (Minuta Tech) was drop-dispensed onto the surface, and a PUA mold containing the engraved nanoscale zigzag patterns was placed directly on each coated surface. The PUA precursor spontaneously moved into the cavity of the mold by means of capillary action and was subsequently cured by exposure to UV light (λ = 250–400 nm, 100 mJ/cm^2^) for ∼30 s through the transparent backplane. After the curing process, the molds were peeled from the surfaces.

### T cell Preparation

DO11.10 T cell receptor transgenic mice were purchased from Jackson Laboratories and bred in the animal care facility in POSTECH Biotech Center (PBC) under pathogen-free conditions. All experiments involving mice were approved by the Institutional Animal Care and Use Committee at PBC. Next, DO11.10 CD4+ T cell blasts were prepared by stimulating cells from the spleens and lymph nodes of DO11.10 T cell receptor transgenic mice with 1 mg/mL OVA323–339 peptide (ISQAVHAAHAEINEAGR, Peptron, Inc. Korea). Then, DO11.10 blasts were cultured in R-10 (RPMI media (Gibco) with 10% fetal bovine serum (Gibco), 100 U/mL penicillin, 100 mg/mL streptomycin (Invitrogen)) with 1–2 U/mL interleukin-2 (Peprotech), and the T cells were used 5–7 days after stimulation.

### Migration Assays

For migration assays, the nanoscale zigzag structured surfaces were coated with 10 µg/mL ICAM-1 (R&D systems) by incubating the ICAM-1 solution in PBS for 1 h at 37°C after about 60 s air plasma treatment (200–500 w, Femto Science, Korea). T cell blasts labeled with 10 µM 5-(and-6)-(((4-chloromethyl)benzoyl)amino)tetramethylrhodamine (CMTMR, Invitrogen) were seeded onto the surfaces, incubated for 2 h, and then imaged by microscope. Ultra-low-melt agarose (USB) at a final concentration of 1% was added to the R-10 medium to minimize convection during live cell imaging. For pharmacological inhibition of Arp2/3, the T cells were treated with 50 µM CK-636 (Sigma). The inhibitor was added to R-10 media containing 1% ultra-low-melt agarose.

### Live Cell Imaging and Data Analysis

A modified Zeiss Axio Observer Z1 epifluorescence microscope with a 40X (Plan-Neofluar, NA = 1.30) objective lens and a Roper Scientific CoolSnap HQ CCD camera was used for imaging. The T cell seeded surfaces were mounted on microscope stage equipped with a Chamlide TC incubator system (Live Cell Instrument, Seoul, Korea) maintaining 37°C and 5% CO_2_ for live cell imaging. Time-lapse microscopy was immediately initiated with images recorded at intervals of 15 s for 15 min. At each time interval, differential interference contrast (DIC) and red fluorescence (EX BP 550/22 and EM BP 605/70) images were recorded in rapid succession for each acquisition. The trajectories of the T cells were analyzed using the ‘track object’ function of Metamorph (Universal Imaging, Molecular Devices).

### Scanning Electron Microscopy

Cells were fixed in PBS with 4% paraformaldehyde and 2% sucrose at room temperature for 20 min. After washing these cells with PBS twice, we fixed them in the second fixative (3% paraformaldehyde and 2.5% glutaraldehyde in 0.1-M cacodylate buffer supplemented with 1% sucrose and 5-mM CaCl_2_, pH 7.4) at room temperature for 20 min. After washing the cells twice with a 0.1-M cacodylate buffer, the cells were dehydrated in a series of ethanol solutions (from 30% to 99.5%) and finally in hexamethydisilazane. After air drying the cells, we sputter coated them with gold and observed them using a Philips XL30S.

## Supporting Information

Movie S1 Representative movie of a T cell ‘crossing’ the interfaces of the nanoscale zigzag structures (L = 60 µm, θ = 45°). Time stamp = min:sec, Scale bar = 20 µm.
(AVI)

Movie S2 Representative movie of a T cell ‘migrating along’ the interfaces of the nanoscale zigzag structures (L = 60 µm, θ = 45°). Time stamp = min:sec, Scale bar = 20 µm.
(AVI)

Movie S3 Representative movie of a CKC-636-treated T cell ‘trapped’ at the interfaces of the nanoscale zigzag structures (L = 60 µm, θ = 45°). Time stamp = min:sec, Scale bar = 20 µm.
(AVI)
